# Online or Offline? How Smog Pollution Affects Customer Channel Choice for Purchasing Fresh Food

**DOI:** 10.3389/fpsyg.2021.682981

**Published:** 2021-05-26

**Authors:** Jing Liang, Jiangshui Ma, Jing Zhu, Xu Jin

**Affiliations:** School of Business Administration, Faculty of Business Administration, Southwestern University of Finance and Economics, Chengdu, China

**Keywords:** e-commerce, customer behavior, channel choice, smog pollution, omnichannel

## Abstract

Due to fresh foods' unique characteristics, where quality, freshness, and perishability are the main concerns, consumers are more inclined to choose offline channels for purchasing foods. However, it is not well-understood how these behaviors are affected by the adverse external environment, e.g., smog pollution. Fine particulate matters (PM2.5) on smog days would irritate the respiratory tract and pose health risks to people, triggering negative emotions such as sadness and depression. People tend to stay in a clean indoor environment on smog days. An adverse external environment is causing a gradual change in people's habits and emotions. Still, its impact on shopping behaviors is a complex process in need of further study. The study fills this gap by examining the impact of smog pollution on customer channel choice. Based on data from an e-commerce retailer that operates in both online and offline channels. We find that (1) the degree of smog pollution has a significant positive effect on online channel purchasing at aggregated store-, product-, and individual- levels; (2) moreover, the retailer's in-store interactive activities would restrain this positive relationship; (3) variation of product pricing and customers' healthy eating tendency would pronounce the positive association between smog and online purchasing. These results can serve as a reference for retailers to adjust channel strategies in the face of harsh external conditions.

## Introduction

Smog is one of the top environmental concerns in China. According to the official data released by the Ministry of Environmental Protection, the annual average PM2.5 (particles <2.5 micrometers in aerodynamic diameter) concentration is 55–130 lg/m^3^ in 2018, far exceed the safety standard (10 lg/m^3^) set by the World Health Organization. Visibility is poor on smog days, and there are lots of dust, pollutants, microorganisms in the air. Smog could result in severe health damages. It is associated with increased mortality, cancer incidence, physician visits, and a significant reduction in life expectancy (Brunekreef and Holgate, 2002).

Air pollution has various effects on social behaviors and production. As noted by Heider (1982), human behavior is subject to internal and external influences, and some of the main external factors are related to environmental and weather conditions. As an external environmental factor, smog pollution would stimulate the respiratory tract, causing cough, suffocation, shortness of breath and other uncomfortable reactions, which restrains the mood of residents and triggers irritability and depression. The emotional states associated with smog pollution can cause gradual changes in diet- and travel-related behaviors, reducing people's willingness to go outside.

**Figure 1:**
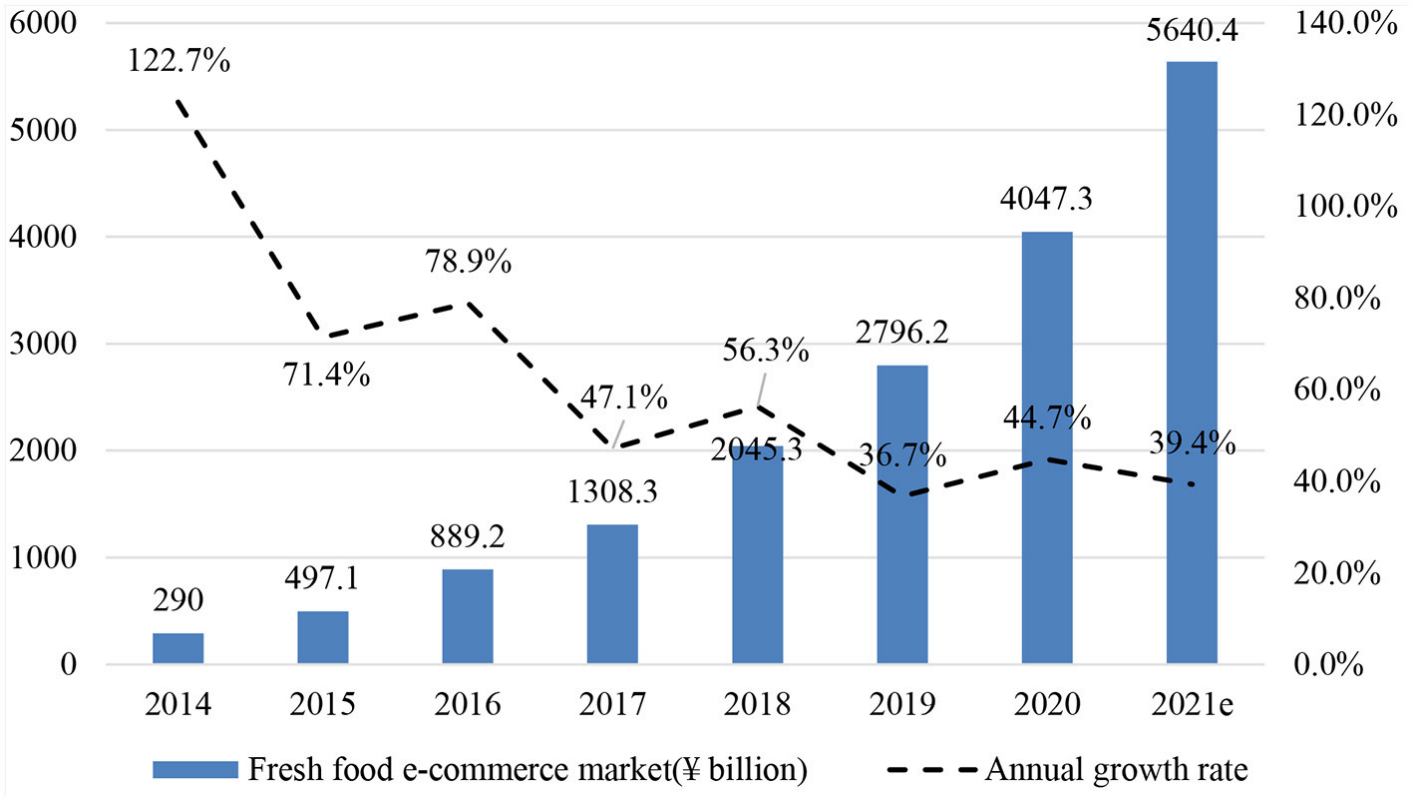
Growth in the Chinese fresh food e-commerce market. Source from iResearch “2020 China's Fresh Food E-commerce Report,” July 13, 2020. Data in 2021 are predicted values estimated by iResearch.

Meanwhile, e-commerce has taken hold in China in recent years. The fresh food e-commerce market, which enables consumers to purchase fresh foods using online channels, has developed rapidly. As shown in Figure 1, the size of China's fresh food e-commerce market was 290 billion (CNY) in 2014 and increased to 4047.3 billion (CNY) in 2020. The annual growth rate of the fresh food e-commerce market shows a fluctuating downward trend, indicating that the market is getting matured. At the same time, sizeable fresh food e-commerce companies, including Amazon Fresh (owned by Amazon), Hema Fresh (owned by Alibaba), and 7FRESH (owned by JD.com), are developing offline stores, mainly because brick-and-mortar stores are still the primary channel for purchasing fresh foods. Given that fresh foods are non-standardized products with short shelf lives, consumers often prefer to judge their quality by observing and physically handling them. Offline channels also have the advantage of satisfying immediate demand; for example, consumers who need to cook immediately tend to purchase ingredients from a near store, as purchasing online requires them to wait for delivery.

However, it is reasonable to expect this preference for offline channels to change in the presence of smog pollution, given that people are likely to go out as little as possible on smog days to avoid adverse health impacts. The desire to stay in clean indoor environments may cause a shift in purchase behaviors toward fresh food by motivating people to opt for online channels. Many marketing researchers studied customer behaviors through omnichannel (Reardon and McCorkle, 2002; Verhoef et al., 2007; Choi and Mattila, 2009; Neslin and Shankar, 2009; Avery et al., 2012; Kleinlercher et al., 2018, 2020; Goraya et al., 2020; Lawry and Bhappu, 2021) and found evidence of cross-channel cannibalization (e.g., Avery et al., 2012). It is important to reveal the external condition under which customers' purchasing behavior would shift from one channel to another. Existing research on the impact of an adverse external environment mainly focused on the fields of economics, psychology, and medicine, with most related studies exploring how air quality affects tourism, transportation, energy consumption, and medical care expenditure. There is limited research on how air pollution affects consumer behavior. Furthermore, most studies rely on questionnaires to examine the effects of smog weather on consumer psychology and behavior, even though results based on this method are easily affected by factors such as differences in subjective judgments. This study adopts a more objective method by using actual transaction/sales data from an omnichannel fresh food retailer to analyze consumer behavior.

This paper studies the impact of smog pollution on consumers' channel selection behavior for purchasing fresh food. It also considers (1) the moderating effect of interactive activities at the store level; (2) the moderating effect of product price volatility at the product level, and (3) the moderating effect of customers' healthy eating tendency at the individual level. We focus on fresh food because it is a very offline-preferred category. First, many customers buy fresh food for daily cooking, and offline shopping can satisfy their demand immediately, while online purchasing needs customers to wait for the delivery at home. Second, the offline channel provides the “feel and touch” experience and on-site service, and customers may decide what to buy for dinner on the spot at the offline shop. Third, the quality guarantee period of fresh food is relatively short, and it needs to be refrigerated or frozen to keep fresh, thus the delivery of fresh food requires high speed and cold chain transportation, bringing challenges for selling it in the online channel. Meanwhile, smog pollution makes people go out less and reduces the frequency of visiting physical stores, which is expected to influence the purchase behavior of such offline-preferred categories.

Based on transaction data from an e-commerce fresh food retailer that owns both online and offline channels, this study finds that the degree of smog pollution has a significant positive effect on the proportion of purchasing fresh food through online channels, and the retailer's in-store interactive activities play a negative moderating role in this effect. In contrast, product price volatility and customers' healthy eating pronounce the positive effect of smog on online channel consumption. These findings enrich the psychological and marketing literature by providing empirical support for the idea that an unfavorable external environment stimulates changes in consumer behavior. This study can provide a reference for retailers to adjust channel strategy effectively under the severe external environment.

This paper is structured as follows. Section Introduction introduces the study background and research questions. Section Literature Review and Hypothesis Development reviews the relevant literature as well as proposes our hypotheses. In section Data and Measurements, we explain data sources, sampling, indicators and empirical models. Section Results presents empirical results and robustness tests. Finally, in section Conclusion and Discussion, we conclude with the discussions, implications and limitations of this study.

## Literature Review and Hypothesis Development

### The Impact of Air Pollution

Air pollution negatively affects people's emotions and activities. The Mehrabian-Russell (M-R) model indicates that environmental stimuli trigger emotional responses and that these emotions, in turn, influence behavior. Environmental stimuli mainly refer to the influence of environmental characteristics on the five human senses. The model proposes that environmental stimuli trigger emotional states along the three basic dimensions of pleasure, arousal, and dominance. Each emotional state is associated with certain behavioral responses, mainly acceptance or avoidance. Evans and Jacobs (1981) found that exposure to a heavily polluted environment aggravates negative emotions such as depression, anxiety, and tension. Similarly, Baumgartner (2002) and Lamers et al. (2011) noted that air pollution is associated with mood disorders and worsening depressive symptoms. Further, Li and Peng (2016) found that depression caused by air pollution can affect an individual's behavior. In terms of consumer activities, Zhang and Mu (2018) documented a significant increase in mask consumption during periods of extreme air pollution, especially masks designed to protect against PM2.5. Kang et al. (2019) empirically tested the relationship between air pollution and retail sales of consumer goods, finding that more severe air pollution was associated with a larger fall in retail sales. In addition, Li et al. (2017) argued that air pollution would have a negative effect on sales of fuel-inefficient cars. Cai and He (2016) proposed that people tend to stay in clean indoor environments and reduce outdoor travel when the weather is hazy due to health concerns. In terms of economic and financial activities, Heyes et al. (2016) found that a one standard deviation increases in PM2.5 in the environment causes a decrease in the rate of return on securities transactions. To sum up, air pollution negatively affects people's emotional states, consumption, outdoor behavior and economic activities.

With respect to the relationship between air pollution and customers' channel selection behavior, we assume that offline shopping will decrease and online purchasing will increase under severe air pollution. Retailing studies have found that the shopping environment (e.g., cleanliness, in-store air quality) affects consumers' purchasing behavior, and declining sales of stores are related to unpleasant circumstances (Kumar and Karande, 2000; Turley and Milliman, 2000; Nicholson et al., 2002; Chocarro et al., 2013; Kang et al., 2019). Meanwhile, evidence has shown that consumers have channel switching behavior among different channels (Reardon and McCorkle, 2002; Choi and Mattila, 2009; Avery et al., 2012; Kleinlercher et al., 2018; Li et al., 2020). Specifically, Reardon and McCorkle (2002) outlined that consumers' channel choices are influenced by five factors: the relative opportunity cost of time, the cost of products, the pleasure of shopping, the perceived value of products and the relative risk of channels. When external conditions are harsh, consumers are less willing to go out and visit physical stores. Exposure to smog pollution increases the risk of respiratory and other diseases and makes people feel anxious and depressed (Power et al., 2015), which adds the risk of offline shopping and reduces the customers' pleasure in visiting physical stores. Thus, customers are likely to change their purchasing channels and switch from offline channels to online channels. Based on the above discussions, we propose the following hypothesis:

***H1***: The proportion of online purchasing for fresh food is positively related to the degree of smog pollution.

### Channel Selection Behavior

Consumers' channel selection behavior is affected by various factors, such as product category characteristics, prices, differentiated services, and consumer preferences. Chocarro et al. (2013) showed that channel selection behavior is influenced by environmental factors, time factors, and social factors. Yang et al. (2013) proposed that the perceived levels of services in different channels affect a consumer's use of a particular channel. Xu and Jackson (2019) indicated that perceived risks have a negative impact on customers' willingness to choose channels. In addition, given that online and offline channels have different characteristics that lead to different consumer experiences, consumers' channel selections are significantly influenced by the kind of shopping experiences they wish to have. Akaah et al. (1995) pointed out that non-store channels are more convenient than offline stores and provide opportunities to save time and effort. Verhoef et al. (2007) studied the consumers' research shopping behaviors, that is, searching for information and purchasing through different channels. They pointed out that cross-channel synergy has promoted this phenomenon. Schröder and Zaharia (2008) noted that consumers' channel selection habits are likely to change in the presence of promotions. In sum, consumers evaluate the costs and benefits of different consumption channels with distinct characteristics, and these evaluations are the main driver of channel migration behavior (Ansari et al., 2008).

Compared with online consumption channels, offline channels are characterized by experience and interactivity. In-store interactive activities offer action and fun, creating additional experience gains for consumers, but they do not necessarily provide specific product-related information (Hede and Kellett, 2011; Leischnig et al., 2011). Retailers can implement in-store interactive activities, turning their stores into places where people can participate and learn in entertainment activities, such as playing music, scrapbooking, painting, and even exercise (Sands et al., 2015). Many studies have found that in-store activities have a positive effect on offline store operations with heterogeneous conditions and degrees. For example, Holmqvist and Lunardo (2015) found that the recreational in-store experience positively influences the pleasure and shopping intentions of those consumers with entertainment motives. Chaney et al. (2016) revealed that in-store activities increase purchasing intentions when consumers were not aware of the goals behind the activities. Using data from 356 questionnaires collected in Indian, Kumar and Polonsky (2019) indicated that retailers' in-store activities led to increased consumers' perceived credibility, and customers' experience quality mediated this relationship. Research also found that holidays and weekends significantly impact consumers' shopping behavior, as merchants tend to concentrate their special events and interactive activities with customers around these times to entice consumers to stores (Smith, 1999; Leszczyc and Timmermans, 2001). Base on the previous literature, we assume that the retailer's in-store interactive activities can attract consumers to visit the store, which could moderate the relationship between external smog pollution and consumer channel choices. Thus, Hypothesis 2 is proposed:

***H2***: Stores' interactive activities have negative moderating effects on the relationship between smog and the proportion of online purchasing for fresh food.

Fresh food prices oscillate because retailers adjust each product's price based on their stocks and costs. For example, strawberries in non-harvest seasons are usually more expensive. We expect that product price fluctuation would influence customers' channel choice behavior. Online channel search can provide consumers with more price information, allowing them to obtain better deals (e.g., Bakos, 1997; Morton et al., 2001; Verhoef et al., 2007). It is easier to compare prices and information through online channels with just clicking a button, while comparing prices offline needs customers to actually explore the shop one by one (Pauwels et al., 2011; Kollmann et al., 2012). Thus, under the same conditions, those products with fluctuating prices are more inclined to be sold through online channels. Mosquera et al. (2018) also suggested that in-store services should offer a mobile app to enhance the purchasing experience through easier price comparison and real-time shock checking services. Many retailers indeed developed their own apps to provide the customers with online search services, such as Costco and Decathlon. Xu and Jackson (2019) indicated that channel advantages (e.g., easier price comparison online) positively affect customer channel choice intention. For products with high price volatility, easier price comparison makes consumers more inclined to choose online channels to purchase these foods. Thus, we propose Hypothesis 3 as:

***H3***: the positive relationship between smog and online purchase is more pronounced for those products with higher price fluctuations.

When smog days, there were lots of dust, pollutants, microorganisms in the air, which would not only restrain the mood of residents but also stimulate the respiratory tract, causing the cough, suffocation, shortness of breath, and other uncomfortable reactions. Smog could result in severe health damages. Some foods, such as fresh fruit, vegetable and seafood, are considered to help mitigate smog's adverse effects. These foods are rich in antioxidants and have anti-inflammatory effects, which could help clean the system, particularly the airway of human beings (Hertog and Hollman, 1996; Polyfenols, 1998). Most of these foods are light and low in calories, usually for those who want to stay healthy or lose weight. We expect consumers who have higher recognition of healthy eating are more sensitive to the adverse effects of smog and care more about how to keep healthy. They are likely to shop online instead of going outside on smog days. Thus, Hypothesis 4 is proposed:

***H4***: Consumers who engage more in healthy eating are more likely to purchase fresh food online when the degree of smog is higher.

## Data and Measurements

### Sample

We study the S E-commerce Fresh Food Company, a membership-based retailer that operates both an online channel and offline stores in Southwest China. It has a wide range of products, including vegetables, fruits, meat, oil, poultry, eggs, seafood, snacks, and some processed food, such as fried and baked meat. Currently, the company has 21 stores in city C and more than 30,000 members.

The retailer's offline stores were opened near its target communities. Those offline stores also served as sub-warehouses or front warehouses of communities, and they were responsible for the delivery for communities within a 2 km radius. Orders were delivered by employees in each store (when there was no online order that needs to be delivered, these employees worked as the store's salespersons and shopping guiders). All the online orders were promised to be delivered within 1 h. The operating condition of this retailer remained consistent from 2014 to 2016. In 2017, the retailer's business underwent a major change. Therefore, our sample only contains data from 2014 to 2016 for maximum comparability.

**Figure 2:**
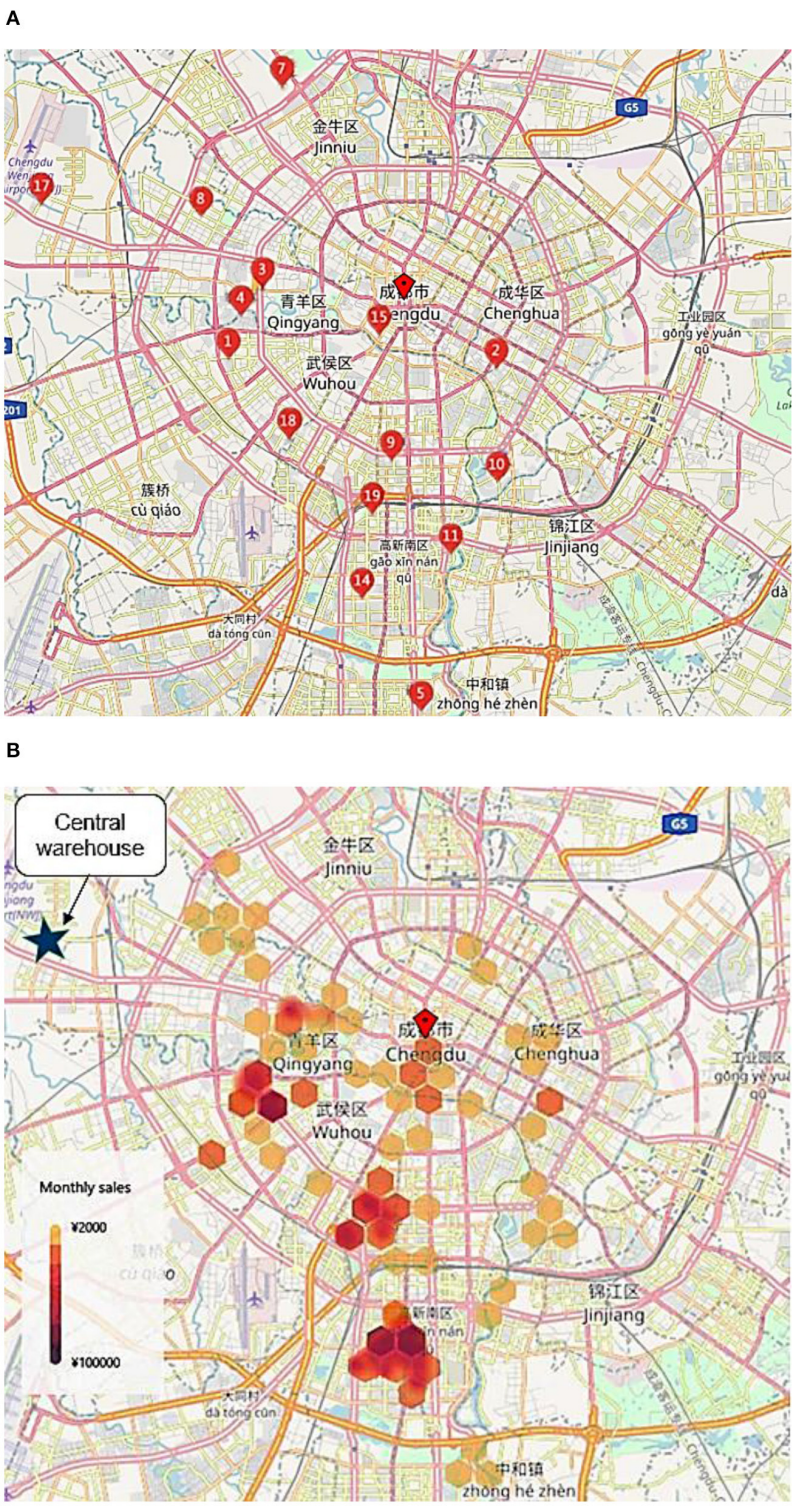
**(A)** Store location of the retailer. **(B)** Heat map of sales distribution. Location 17 is the central warehouse. The other locations are offline shops in city C.

We choose this retailer as our research object because it allows us to estimate the effect at multiple levels, which perfectly matches our research needs. First, the company owns both online channels and offline physical stores, which basically cover city C's residential areas. People in these communities choose the nearest shop when they purchase offline for the daily meal. As shown in Figure 2, monthly total sales mainly revolve around offline stores, which allows us to measure the proportion of online and offline sales at the location-aggregated level. Second, the retailer provides the same category of products in each channel and store, which allows us to calculate the proportion of online and offline sales at the product level. Third, it sells fresh food based on a membership system; as customers need to register as members before making purchases, the full transaction history for each person is available. This allows us to explore the relationship at the individual level.

To eliminate bias caused by enterprise purchasing and other extreme conditions, we identify and remove purchases that do not represent daily consumer behavior. Specifically, we exclude all order data with purchase quantities >99 since these orders essentially represent corporate purchases, according to the interview with the retailer manager. We also drop those transactions and customer IDs that only purchase once during the sample period because these are likely to be casual shopping, not long-term behavior. Statistical analysis reveals that each consumer has an average consumption record of about 64 days.

Altogether, our sample contains 2,239,530 transaction records from 17,467 unique members from January 2014 to September 2016. The sample comprises 1,562 different products. Each transaction includes member ID, order ID, order date, which channel, names, quantity, price and SKUs of products purchased, from which we can calculate the proportion of online and offline sales and other variables we need.

### Measurements

#### Dependent Variables

The dependent variables are measured based on the online channel sales and the proportion of online purchase payments out of total purchase payments at three different levels. To be specific, the store-level proportion is measured as the store *i*'s online sales at *t* day divided by its total sales, denoted by *Store_Online*_*it*_. Similarly, the product-level proportion is calculated as the product *j*'s online sales divided by the total sales of this product through omnichannel at *t* day, denoted by *Product_Online*_*jt*_.

Customers usually use one channel to buy things in a day. For example, a customer who buys foods online is likely not to go shopping offline again that day. Therefore, we measure the individual-level online purchase proportion on a weekly basis, *Individual_Online*_*dw*_, calculated as customer *d*'s online channel purchases divided by the total purchases during *w* week.

#### Independent Variables

We use the daily PM2.5 concentration of city C as a proxy for the degree of smog pollution, denoted as *PM2.5*_*t*_. Smog is generally caused by high concentrations of fine particles in the air. This particle's size is less than or equal to 2.5 btm, referred to as PM2.5 (Xing et al., 2016). It can be suspended in the air for a long time. PM2.5 has a strong activity, which is easy to attach toxic and harmful substances (such as heavy metals, microorganisms), and has an essential impact on visibility. We use PM2.5 concentration data published by the U.S. Embassy in Chengdu^1^ to measure the daily smog magnitude from January 2014 to September 2016.

For robustness testing, we use the degree of the air quality index (AQI) as an alternative indicator for PM2.5. The daily AQI is provided by the Ministry of Environmental Protection and is the most common index used for measuring air quality in China. AQI is calculated based on the concentration of primary pollutants that cause air pollution, including fine particulate matters, respirable particulate matters, sulfur dioxide, nitrogen dioxide, ozone, and carbon monoxide. The AQI is generally divided into six levels: Grade I (Excellent), Grade II (Good), Grade III (Light Pollution), Grade IV (Medium Pollution), Grade V (Heavy Pollution), and Grade VI (Extremely Heavy Pollution). If air quality is more than Grade II, it is considered to be harmful to health. We label the days with air quality more than Grade II as “smog day” since it might be harmful to health when air quality is higher than Grade II.

**Figure 3:**
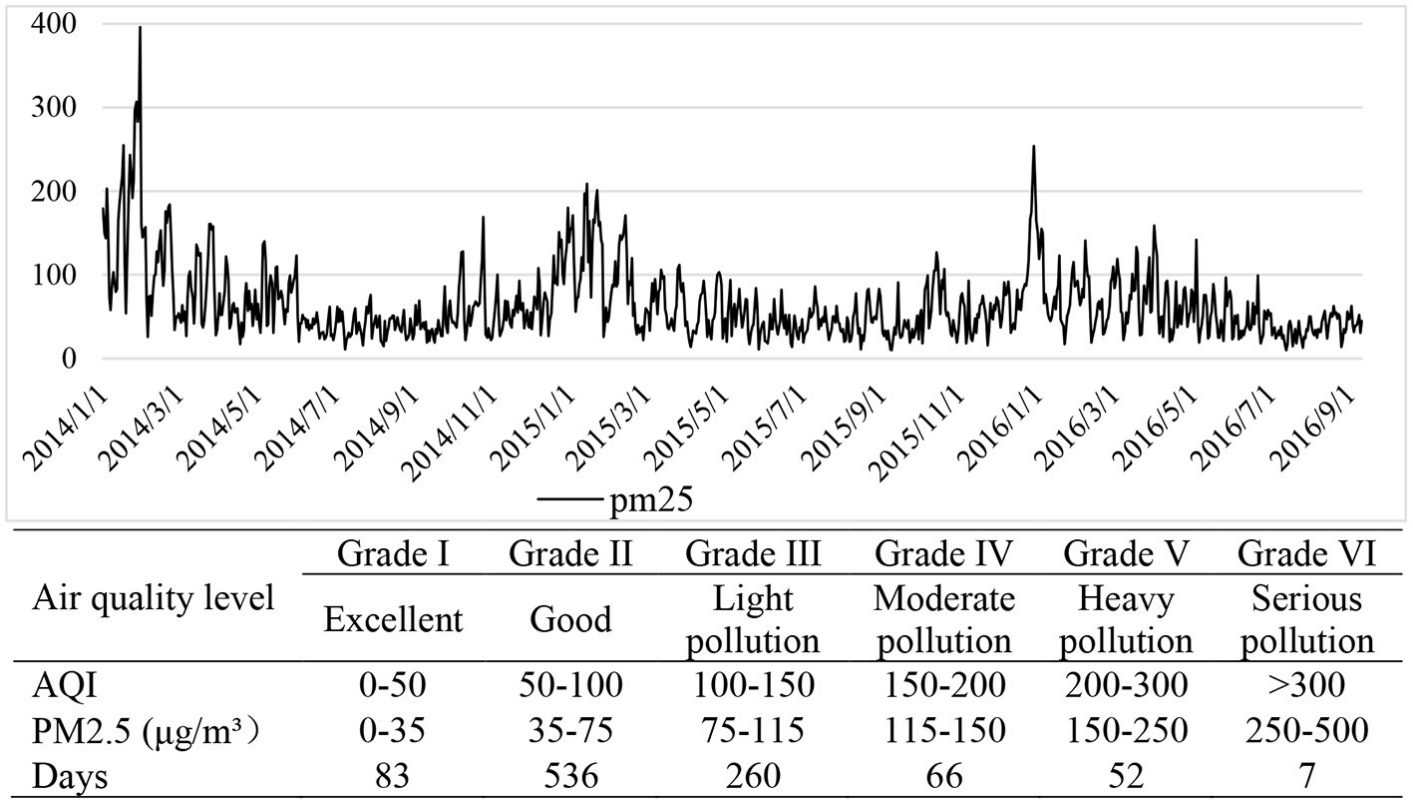
The air quality of city C during the sample period.

As shown in Figure 3, we can see that smog is seasonal. The air condition is more severe in winter (especially in January). Thus, we add season dummies in the regression model to control for seasonality. During the sample period, air quality is rated as excellent or good in about 61.65% of total days. The number of smog days is 385, accounting for about 38.35% of the days during the sample period.

#### Moderators

Based on our hypotheses, we construct moderators at three-level. First, the retailer's interactive activities might influence customers' cross-channel purchasing behavior. We use a dummy variable *Activities*_*it*_ to indicate store *i*'s in-store interactive activities, which equals 1 when the retailer conducts in-store interactive activities at *t* day and 0 otherwise.

Second, we measure the product price volatility by calculating the covariance of price for each product as the variation of the product's price divided by the mean of the product's price, denoted by *Covariance_Price*_*jt*_. Using the covariance allows us to avoid the bias brought by price heterogeneity.

Third, customers who are concerned about healthy eating (e.g., those who buy more healthy food on smog days) are more likely to shop online when the external environment is severe. We count the healthy food proportion for each order, denoted by *Healthy_eating*_*dw*_, to proxy this indicator. Research has shown that vegetables, fruits, olive oil, and seafood rich in vitamins C, E, and A and polyphenols exhibit anti-tumorigenic, anti-mutagenic anti-inflammatory, and antiviral actions (Hertog and Hollman, 1996; Bravo, 1998). We divide food into two categories: anti-smog foods and other food. The anti-smog foods group includes *vegetables, fruits, olive oil*, and *seafood*, as they can help keep healthy on smog days. On the other hand, other foods are foods characterized by general eating, such as meats, poultry, snacks, fried and baked goods, eggs, and *rice*.

#### Controls

We construct control variables also at three levels. First, each store's business conditions might impact customers' online shopping behavior, so we measure the store-level condition as the total daily sales of the store that the order belongs to, denoted by *Store_amount*_*it*_. Second, the demands of various products are diverse. A customer may buy vegetables every day but purchase rice only once a month. Therefore, we use each product's daily sales to control the heterogeneous product needs, denoted by *Product_amount*_*jt*_. Third, customers' eating habits, shopping habits and financial situation might also impact their channel choice behaviors. We calculate the total amount of customer *d*' orders during *w* week to represent the customer's heterogeneity, denoted by *Order_amount*_*dw*_. Last, we compute the retail's daily sales in total, controlling for the general impact on the sample, denoted by *Total_sales*_*t*_.

To control for heterogeneity of stores, products and individuals. Store dummies, product dummies, and individual dummies are also included when estimating the relationship between PM2.5 and online purchase proportion at aggregated store level, aggregated product level, and individual level, respectively. In addition, as food consumption behaviors vary across seasons, we include season dummies to control seasonal effects. The descriptions of key variables are listed in Table 1.

**Table 1:** Variable definitions.

**Variable**	**Descriptions**
**Dependent variables**	
*Store_Online_*it*_*	The proportion of online sales on total sales of store *i* at *t* day, which is measured as the store's online channel sales divided by its omnichannel sales.
*Product_Online_*jt*_*	The proportion of online sales on total sales of product *j* at *t* day, which is measured as the product's online channel sales divided by its omnichannel sales.
*Individual_Online_*dw*_*	The individual-level online purchase proportion, which is measured as the customer *d*'s online purchases divided by total purchases during week *w*.
**Independent variables**	
*PM2.5_*t*_*	Daily PM2.5 concentration data (μg/m^3^)
Air quality degrees	Air quality index (AQI) levels (0 = Excellent; 1 = Good; 2 = Light Pollution; 3 = Medium Pollution; 4 = Heavy Pollution; 5 = Extremely Heavy Pollution)
**Moderators**	
Activities_it_	A dummy variable that equals 1 when there are in-store interactive activities at *t* day and 0 otherwise.
*Covariance_Price_*jt*_*	The variation of the product's price is divided by the mean of the product's price.
*Healthy_eating_*dw*_*	Each customer's healthy food proportion in purchasing order.
**Controls**	
*Store_amount_*it*_*	The total sales of store *i* at *t* day.
*Product_amount_*jt*_*	The total sales of product *j* at *t* day.
*Order_amount_*dw*_*	The total amount of customer *d*' orders during *w* week.
*Total_sales_*jt*_*	The daily sales of S company.

### Economic Models

We develop interaction effect models to test the hypotheses at the store- product- and individual levels, respectively. First, we test the relationship between local smog magnitude (*PM*25_*t*_) and consumers' channel choice (*Y*_*it*_) by the reduced-form model to verify hypothesis 1. Then, we estimate complete models to study how smog magnitude and consumers' channel choice interact with moderators, conducting the test for hypothesis 2, hypothesis 3, and hypotheses 4.

Store-level:

(1) $$\begin{array}{l} Y_{it}=\beta_{0}+\beta_{1}{PM25}_{t}+\beta_{2}Promotion_{it}+\beta_{3}{PM25}_{t}\\ \;\times Promotion_{it}+\beta_{4}{Store\text{\_}amount}_{it}+\beta_{5}{Total\text{\_}sales}_{t}\\ \;+Seasonal\;dummy+\alpha_{i}+\varepsilon_{it} \end{array}$$

Product-level:

(2) $$\begin{array}{l} Y_{jt}=\beta_{0}+\beta_{1}{PM25}_{t}+\beta_{2}{Covariance\text{\_}Price}_{jt}\\ \;+\beta_{3}{PM25}_{t}\times {Covariance\text{\_}Price}_{jt}+\beta_{4}{Product\text{\_}amount}_{jt}\\ \;+\beta_{5}{Total\text{\_}sales}_{t}+Seasonal\;dummy+\alpha_{j}+\varepsilon_{jt} \end{array}$$

Individual-level:

(3) $$\begin{array}{l} Y_{dw}=\beta_{0}+\beta_{1}{PM25}_{w}+\beta_{2}{Healthy\text{\_}eating}_{dw}\\ \;+\beta_{3}{PM25}_{t}\times {Healthy\text{\_}eating}_{dw}+\beta_{4}{Order\text{\_}amount}_{dw}\\ \;+\beta_{5}{Total\text{\_}sales}_{t}+Seasonal\;dummy+\alpha_{d}+\varepsilon_{dw} \end{array}$$

*i, j, d, t, w* indicate stores, products, customers, days, and weeks, respectively. ε is the regression residual. The interaction item is a product of *mean-centered* variables. *Y* is a set of indicators representing consumers' online shopping behavior, including *Store_Online*_*it*_, *Product_Online*_*jt*_, *Individual_Online*_*dw*_. Please refer to Table 1 for variable definitions. Equation (1) is the complete interaction model at the store level, where *Promotion*_*it*_ is the store-level moderator and α_*i*_ is the store dummy control for stores' time-invariant characteristics. Equation (2) is the complete interaction model at the product level, where *Covariance*_*Price*_*jt*_ is the product-level moderator, and α_*j*_ is the product dummy control for products' time-invariant characteristics. Equation (3) is the complete interaction model at the individual level, where *Healthy*_*eating*_*dw*_ is the moderator and α_*d*_ is the individual effect control for individual time-invariant characteristics and unobservable heterogeneity. Seasonal dummies are included to control for seasonality. We use the *reghdfe* command in Stata to estimate our economic models, absorbing multiple levels of fixed effects.

## Results

### Descriptive Statistics and Tests

Table 2 reports descriptive statistics as well as the correlations of the variables that are included in the regression. As shown in Panel (B), the correlations of the independent variable, moderators and dependent variables are low, reducing the concern of collinearity. We also calculate independent variables' variance inflation factor (VIF) for each economic model. The values are between 1.00 and 3.61, much <10, also alleviating concerns about coefficient collinearity.

Table 2Descriptive statistics and correlations.

**Table:** 

**Panel (A)** Descriptive statistics of key variables						
**Variables**	**N**	**Mean**	**St.Dev**	**Min**	**Median**	**Max**
*Store_Online (%)*	2,239,530	56.21	21.81	0	56.27	100
*Product_Online (%)*	2,239,530	56.09	26.87	0	56.91	100
*Individual_Online (%)*	2,239,530	54.39	46.35	0	72.08	100
*PM2.5 (μg/m3)*	2,239,530	57.21	35.06	10	49	396
*Store_amount (CNY)*	2,239,530	26,196.43	28,840.96	0	18,329.93	380,014.3
*Product_amount (CNY)*	2,239,530	859.07	2,636.18	0	364.9	105,975.9
*Order_amount (CNY)*	2,239,530	1,728.39	13,787.12	0.04	384.8	270,538.3
*Total_sales (CNY)*	2,239,530	95,881.39	50,010.6	174	87,654.27	451,840.1
*Activities*	2,239,530	0.05	0.22	0	0	1
*Covariance_Price*	2,189,277	0.28	0.25	0	0.22	1.41
*Healthy_eating (%)*	2,239,530	53	28	0	53	100

**Table:** 

**Panel (B) Pearson's correlations**											
**Variables**	**(1)**	**(2)**	**(3)**	**(4)**	**(5)**	**(6)**	**(7)**	**(8)**	**(9)**	**(10)**	**(11)**
**Dependent variables**											
(1) *Store_Online*	1.00										
(2) *Product_Online*	0.37^*^	1.00									
(3) *Individual_Online*	0.44^*^	0.48^*^	1.00								
**Independent variable**											
(4) *PM2.5*	0.08^*^	0.05^*^	0.03^*^	1.00							
**Controls**											
(5) *Store_amount*	−0.25^*^	−0.03^*^	−0.14^*^	−0.08^*^	1.00						
(6) *Product_amount*	−0.05^*^	−0.06^*^	−0.03^*^	−0.04^*^	0.12^*^	1.00					
(7) *Order_amount*	0.11^*^	0.08^*^	0.06^*^	−0.02^*^	0.63^*^	0.11^*^	1.00				
(8) *Total_sales*	−0.13^*^	−0.07^*^	−0.04^*^	−0.20^*^	0.57^*^	0.18^*^	0.38^*^	1.00			
**Moderators**											
(9) *Activities*	−0.14^*^	−0.20^*^	−0.19^*^	−0.05^*^	0.10^*^	0.03^*^	−0.02^*^	0.17^*^	1.00		
(10) *Covariance_Price*	0.00	0.02^*^	−0.01^*^	0.01^*^	0.03^*^	−0.04^*^	0.01^*^	0.02^*^	0.15^*^	1.00	
(11) *Healthy_eating*	−0.01^*^	−0.03^*^	−0.04^*^	−0.03^*^	0.03^*^	0.05^*^	0.00^*^	−0.01^*^	0.01^*^	−0.00^*^	1.00

*In Panel (A), parentheses are the variable units*;

* *denote significance at the level of 0.01*.

In analyses of daily economic behaviors, variable data are often non-stationary. Suppose these non-stationary time series data are directly subjected to regression analysis without cointegration. In that case, there can be undesirable consequences such as spurious regression. Therefore, only by first performing a cointegration test based on a panel data stationarity test can regression analysis guarantee the credibility of the measurement results and related conclusions.

First, to avoid possible errors from using a single test method, three-panel unit root test methods—Fisher-ADF, Fisher-PP, and LLC—are used to test each variable. The results are shown in Table 3.

**Table 3:** Stationarity test of key variables.

**Variables**	**LLC (*P*-value)**	**Fisher-ADF chi-square (*P*-value)**	**Fisher-PP chi-square (*P*-value)**	**Critical result**
*Store_Online*	<0.001	<0.001	<0.001	Smooth
*Product_Online*	<0.001	<0.001	<0.001	Smooth
*Individual_Online*	<0.001	<0.001	<0.001	Smooth
*PM2.5*	<0.001	<0.001	<0.001	Smooth

From Table 3, we can see that the original sequences of all variables are I(0) stationary sequences of the same order.

Next, a cointegration test is carried out to verify whether the original sequence is stable. If the cointegration relationship is established, then regression analysis can be performed on the panel data. Applying the Kao test (McCoskey and Kao, 1998), we perform the cointegration test for each equation.

Table 4 shows that there is indeed a long-term stable cointegration relationship among the variables. Next, the Hausman test method is used to determine whether a fixed-effects model or a random-effects model should be used. The test results are shown in Table 5.

**Table 4:** Results of cointegration test.

**Testing method**	**Hypothesis test**	**Statistical test**	**Equation**	**T statistic value (*P*-value)**
Kao test	H0:ρ = 1	ADF	(1)	−31.6023^***^
			(2)	−34.1375^***^
			(3)	−30.1517^***^

***, **, * *indicate significance at the 0.01, 0.05, and 0.10 level*.

**Table 5:** Hausman test results.

**Equation**	**Chi-square statistic value**	**Critical value**	**Judgment (fixed or random)**
(1)	528.78^***^		Fixed effect
(2)	528.25^***^		Fixed effect
(3)	570.36^***^		Fixed effect

***, **, * *indicate significance at the 0.01, 0.05, and 0.10 level*.

Combining the F test results and the Hausman test, this paper can finally determine that the research model is more suitable for conducting a fixed-effects model. The corresponding regression analysis can be carried out on this basis. As we carry out the empirical study at three different levels, the *reghdfe* command in Stata is applying to estimate our economic models absorbing multiple levels of fixed effects.

### Model Estimates

Table 6 presents the estimation results of Equations (1–3). We first test a reduced-mode model focusing on the main effect of *PM2.5* on the proportion of online purchases. Columns (1) through (3) in Table 6 shows that the significant positive association between *PM2.5* and the proportion of online purchases is robust at the store-, the product- and the individual- levels. An increase of one unit of smog will increase the online sales proportion of the store by 0.03% (β = 0.0327, *p* < 0.01), the average online sales proportion of each product by 0.03% (β = 0.0259, *p* < 0.01), and the *weekly* online orders of each consumer by 0.03% (β = 0.0292, *p* < 0.01). The results provide strong support to ***H1***, indicating that the proportion of online purchasing for fresh food is positively related to the degree of smog pollution.

**Table 6:** Main effect regression results.

**Dependent variable:**	**Predicted Sign**	**(1)**	**(2)**	**(3)**
		***Store_Online***	***Product_Online***	***Individual_Online***
*PM2.5*	+	0.0327^***^	0.0259^***^	0.0292^***^
		(4.57)	(9.45)	(7.16)
*Store_amount*		0.0002		
		(1.55)		
*Product_amount*			−0.0000	
			(−0.32)	
*Order_amount*				−0.0011^**^
				(−2.10)
*Total_sales*		−0.0001^***^	−0.0000^***^	−0.0001^***^
		(−3.24)	(−4.55)	(−9.98)
Season 2		−0.4565	−0.4729	0.8965^**^
		(−0.44)	(−0.90)	(2.50)
Season 3		1.1321	0.6397	1.5090^***^
		(0.76)	(1.08)	(3.42)
Season 4		1.7258	1.9995^***^	1.1334^***^
		(1.56)	(3.96)	(2.83)
Constant		61.9613^***^	56.1919^***^	58.7483^***^
		(17.43)	(103.08)	(60.97)
Absorbed FE level		Store	Product	Customer
Categories		21	1562	17467
Observations		2,239,530	2,239,530	2,236,334
Adjusted R-squared		0.403	0.225	0.668

*Columns (1–3) list the estimates for reduced-mode of Equations (1–3), respectively. The regressions that are absorbing multiple levels of fixed effects are calculated by the reghdfe command in Stata. The robust t-statistics are presented in the parentheses, based on standard errors clustered by absorbed FE level*.

***, **, * *indicate significance at the 0.01, 0.05, and 0.10 level, respectively. Please refer to Table 1 for variable definitions*.

**Figure 4:**
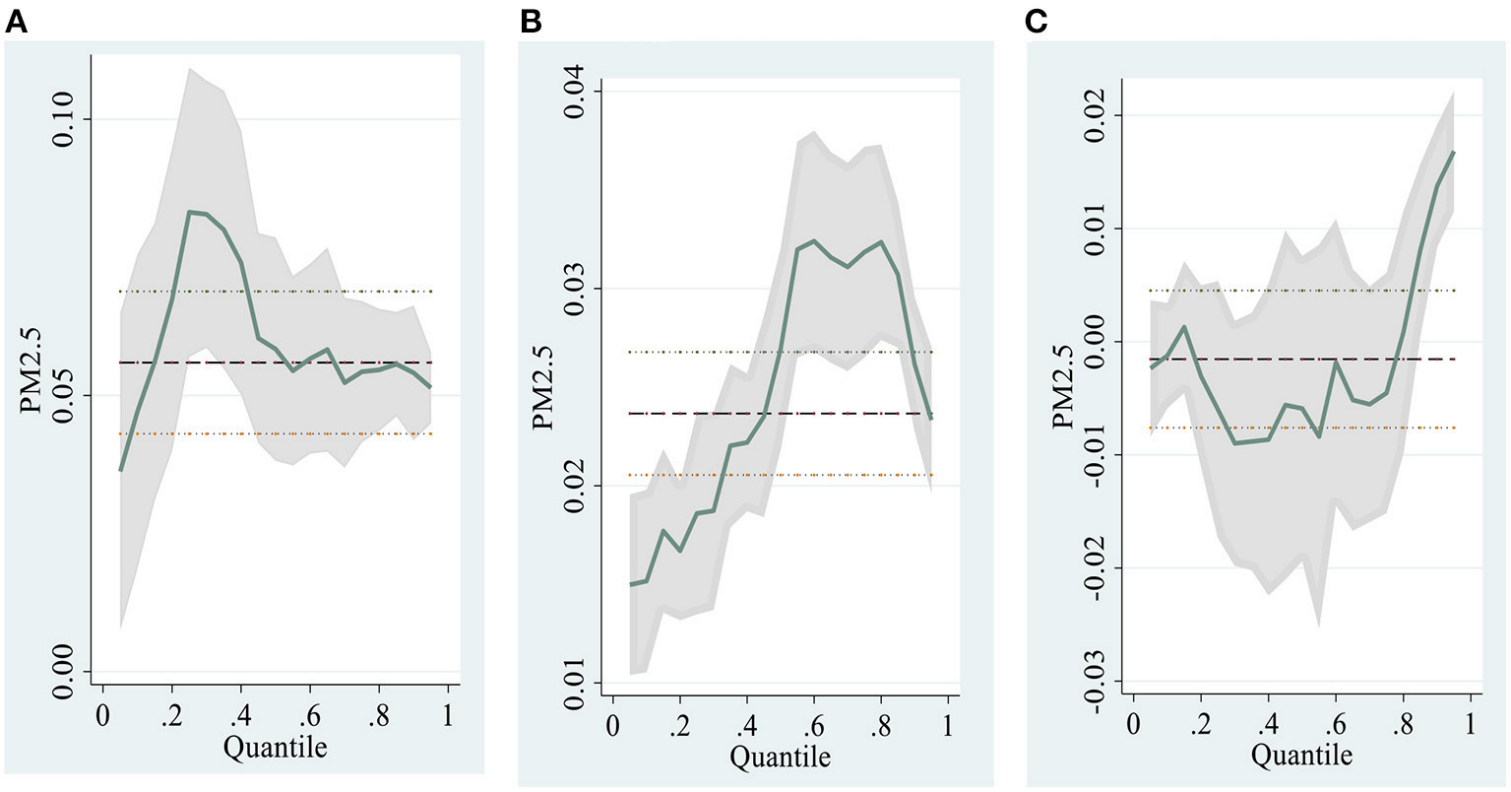
Charts for quantile regression results. **(A)** Store-level **(B)** Product-level **(C)** Individual-level.

With respect to the change magnitude, the coefficient on the PM2.5 is 0.0327 at the store level (Table 6 Column 1), suggesting that a unit increase in PM2.5 magnitude can increase the proportion of online purchases by 0.03%. From Figure 4, we can see that the daily PM2.5 fluctuates significantly. For example, the PM2.5 index was 57 on March 31, 2016, and increased to 159 on April 1, 2016. This change in PM2.5 led to an increase in the proportion of online purchases by 3.06%. In addition, by combining the coefficient and the standard deviation of PM2.5 at 35.06 (Panel A in Table 2), we can infer that the average daily fluctuation of PM2.5 will bring an increase or decrease of 1.07 percentage points in the proportion of online purchases.

Table 7 shows the estimated results of the complete-mode Equations (1–3). The coefficient estimates of *PM2.5* are still positive and significant, indicating our main effects results are robust across different model specifications. The coefficient estimate of the interaction item *Activities* × *PM2.5* is significantly negative (β = −0.1009, *p* < 0.01), which supports ***H2***, indicating that the in-store interactive activities lessen the positive association between smog and online purchase proportion. In line with ***H3***, the coefficient estimate of the interaction item *Covariance_Price* × *PM2.5* is significantly positive (β = 0.0259, *p* < 0.05), indicating the positive association between smog and online purchase proportion is more pronounced for products with higher price volatility. Consistent with ***H4***, the coefficient estimate of the interaction item *Healthy_eating* × *PM2.5* is significantly positive (β = 0.0163, *p* < 0.1), suggesting the positive relationship between smog and online purchase proportion is more pronounced for customers that are more involved in healthy eating.

**Table 7:** Interaction effect regression results.

**Dependent variable:**	**Predicted Sign**	**(1)**	**(2)**	**(3)**
		***Store_Online***	***Product_Online***	***Individual_Online***
*PM2.5*	+	0.0307^***^	0.0264^***^	0.0172^***^
		(4.19)	(9.34)	(6.83)
*Activities*		−7.0629^***^		
		(−7.58)		
*Activities × PM2.5*	−	−0.1009^***^		
		(−7.14)		
*Store_amount*		0.0001		
		(1.58)		
*Covariance_Price*			2.1995^**^	
			(2.57)	
*Covariance_Price × PM2.5*	+		0.0259^**^	
			(2.49)	
*Product_amount*			−0.0000	
			(−0.05)	
*Healthy_eating*				−0.0003
				(−0.06)
*Healthy_eating × PM2.5*	+			0.0159^*^
				(1.71)
*Order_amount*				−0.0011^**^
				(−2.08)
*Total_sales*		−0.0001^***^	−0.0000^***^	−0.0001^***^
		(−3.17)	(−4.62)	(−10.02)
Season 2		−0.2948	−0.5629	0.4979
		(−0.28)	(−1.04)	(1.43)
Season 3		1.2634	0.4952	0.9668^**^
		(0.83)	(0.80)	(2.22)
Season 4		1.8222	1.8125^***^	0.8985^**^
		(1.61)	(3.54)	(2.23)
Constant		61.8688^***^	55.6041^***^	59.8310^***^
		(18.01)	(90.17)	(56.32)
Absorbed FE level		Store	Product	Customer
Categories		21	1,562	17,467
Observations		2,239,530	2,189,276	2,236,334
Adjusted R-squared		0.408	0.233	0.668

*Columns (1–3) list the estimates for complete-mode of Equations (1–3), respectively. The regressions that are absorbing multiple levels of fixed effects are calculated by the reghdfe command in Stata. The robust t-statistics are presented in the parentheses, based on standard errors clustered by absorbed FE level*.

***, **, * *indicate significance at the 0.01, 0.05, and 0.10 level, respectively. Variables are mean-centered in the interaction item. Please refer to Table 1 for variable definitions*.

Note that the coefficient of *Healthy_eating* is not significant (β = −0.0486, *p* > 0.1), indicating that customers' healthy eating habit does not impact their tendency to purchase through the online channel. However, the significantly positive coefficient of *Healthy_eating* × *PM2.5* indicates that when there is smog, healthy-eating customers are more inclined to stay at home and purchase online. The marginal effect of PM2.5 magnitude can be calculated as 0.0172 + 0.0159 × *Healthy_eating*. It implies that an increase in *Healthy_eating* by one standard deviation (28) is associated with a relative increase of 0.4452 in the effect of PM2.5 on online purchase proportion. This result suggests that people who have healthy eating habits will be more sensitive to the adverse effects of smog on their health, so they are more likely to stay in clean rooms and use online channels to buy food on smog days.

### Subsample Tests

Figure 1 in the introduction section shows the size of China's fresh food e-commerce market was growing year by year, which indicates that a larger share of people started to have the experience of purchasing fresh food online. We retest the relationship of PM2.5 and online purchases proportion by year to reveal the possible time tend of our findings, and the results are listed in Table 8.

**Table 8:** Subsample results by year.

**Dependent variable:**	******Store_Online******			***Product_Online***			***Individual_Online***		
	**(1)**	**(2)**	**(3)**	**(4)**	**(5)**	**(6)**	**(7)**	**(8)**	**(9)**
	**2014**	**2015**	**2016**	**2014**	**2015**	**2016**	**2014**	**2015**	**2016**
*PM2.5*	0.020^*^	0.038^***^	0.049^***^	0.019^***^	0.030^***^	0.012^**^	0.015^***^	0.018^***^	0.024^***^
	(2.24)	(3.15)	(2.80)	(6.19)	(7.11)	(2.30)	(2.74)	(2.86)	(3.40)
*Store_amount*	−0.000	0.000^***^	0.000						
	(−0.32)	(4.19)	(0.89)						
*Product_amount*				0.002^***^	0.000	−0.000			
				(2.83)	(0.12)	(−1.17)			
*Order_amount*							0.000	−0.001^*^	−0.001
							(0.94)	(−1.82)	(−1.47)
*Total_sales*	0.000	−0.000	−0.000	0.000^*^	0.000^***^	−0.000^*^	−0.000^***^	−0.000^**^	0.000
	(1.49)	(−1.60)	(−1.35)	(1.69)	(13.68)	(−1.90)	(−4.09)	(−2.53)	(0.44)
Constant	67.969^***^	51.509^***^	47.945^***^	67.096^***^	44.508^***^	52.534^***^	69.976^***^	55.022^***^	49.755^***^
	(46.32)	(23.28)	(28.96)	(131.73)	(83.58)	(75.70)	(120.03)	(25.35)	(66.80)
Absorbed FE level	Store	Store	Store	Product	Product	Product	Customer	Customer	Customer
Categories	21	21	21	1562	1562	1562	17467	17467	17467
Observations	455,712	849,048	934,770	455,694	848,975	934,701	455,271	847,543	933,047
Adjusted *R*2	0.522	0.572	0.622	0.214	0.271	0.324	0.791	0.753	0.766

*Columns (1–3) list the estimates at store level for each year. Columns (4–6) list the estimates at the product level for each year. Columns (7–9) list the estimates at the individual level for each year. The regressions that are absorbing multiple levels of fixed effects are calculated by the reghdfe command in Stata. The robust t-statistics are presented in the parentheses, based on standard errors clustered by absorbed FE level*.

***, **, * *indicate significance at the 0.01, 0.05, and 0.10 level, respectively. Please refer to Table 1 for variable definitions*.

Results in Table 8 indicate that PM2.5 has a continuous positive effect on the proportion of online purchases at the store-, the product- and the individual- levels from 2014 to 2016, consistent with our main findings and address the concern about possible time heterogeneity.

Moreover, from 2014 to 2016, the PM2.5 coefficient shows an upward trend at the store- and individual- levels. With the popularity of online shopping year by year, more and more people have shopped online at least once in their life. This experience makes it easier for them to switch to online channels to buy fresh food when the external smog pollution is severe.

### Robustness Tests

The robustness tests were conducted from three aspects. First, from the results in Tables 6, 7, we can see that the main effect of PM2.5 on online purchases proportion is consistent across reduced-form and complete-form models. We also changed the controls by adding and removing variables and re-estimate the model, showing a similar finding.

Second, we applied the air quality degrees as an alternative indicator of smog levels and reran the model. Estimates are listed in Table 9 and show a positive relationship between air quality degrees and online purchase proportion at the store-, the product-, and the individual- levels, indicating that our main result is solid by using alternative measures.

**Table 9:** Main effect regression results.

**Dependent variable:**	**Predicted Sign**	**(1)**	**(2)**	**(3)**
		***Store_Online***	***Product_Online***	***Individual_Online***
Air quality degrees	+	0.9989^***^	0.6222^***^	0.3280^***^
		(5.48)	(16.75)	(3.62)
*Store_amount*		0.0002		
		(1.56)		
*Product_amount*			−0.0000	
			(−0.27)	
*Order_amount*				−0.0011^**^
				(−2.09)
*Total_sales*		−0.0001^***^	−0.0000	−0.0001^***^
		(−3.35)	(−0.58)	(−10.08)
Season 2		−0.6644	−0.7607	0.2085
		(−0.62)	(−0.84)	(0.60)
Season 3		−0.1542	−0.3406	0.5756
		(−0.10)	(−0.24)	(1.33)
Season 4		1.6565	1.8171^***^	0.7537^*^
		(1.52)	(3.43)	(1.87)
Constant		63.2521^***^	57.3188^***^	60.7286^***^
		(17.33)	(25.35)	(62.95)
Absorbed FE level		Store	Product	Customer
Categories		21	1562	17467
Observations		2,239,530	2,239,530	2,239,530
Adjusted R-squared		0.385	0.221	0.668

*Column (1–3) list the estimates for reduced-mode of Equation (1–3), respectively. Air quality degrees represent air quality index (AQI) levels (0 = Excellent; 1 = Good; 2 = Light Pollution; 3 = Medium Pollution; 4 = Heavy Pollution; 5 = Extremely Heavy Pollution). The regressions that are absorbing multiple levels of fixed effects are calculated by the reghdfe command in Stata. The robust t-statistics are presented in the parentheses, based on standard errors clustered by absorbed FE level*.

***, **, * *indicate significance at the 0.01, 0.05, and 0.10 level, respectively*.

Third, we apply a different estimate method, Quantile Regression (QR), to test the main effect. The panel regression with fixed effects in section Model Estimates focuses on the average relationship of PM2.5 and online purchases proportion, which is susceptible to extreme values. Only considering the average effect might lead to an erroneous regression result. Thus, we use the QR method to explore the coefficient of PM2.5 at every quantile of the online purchases quantiles, and the results are shown in Figure 4.

Figure 4 shows that even though the variation of PM2.5 coefficient is heterogeneous at different aggregated levels, on the whole, the PM2.5 coefficient on each quantile of online purchase proportion is larger than 0, thus, supporting our main findings.

## Conclusion and Discussion

This paper explores how the degree of smog pollution affects consumers' choice of consumption channel, as well as how this effect is moderated by interactive activities, product price fluctuations and customers' healthy eating habits. Overall, smog pollution has a significant positive effect on consumers' tendency to choose online channels, and store interactive activities play negative moderating roles in this relationship. Besides, the positive relationship between smog magnitudes and online purchase proportion is more pronounced for products with higher price variation and customers who have more healthy eating habits. In contrast to previous studies that rely on theoretical approaches, this research is based on real-time consumer transaction data and thus adds insights from a new empirical perspective.

Our findings have important implications for retailers. First, when smog occurs, people tend to go out as little as possible to protect their physical and mental well-being. The desire to stay in clean indoor environments causes a shift in purchase behaviors toward fresh food by motivating people to opt for online channels. The proportion of online purchases increases accordingly. Unlike offline channels, which stimulate purchases through the touch-and-feel experience in the physical store, online channels can utilize various forms of media (e.g., pictures, sounds, video) to provide a full range of product displays stimulate purchases. Retailers can offer information and tips about food functions on the online product description, helping customers choose functional foods to maintain their health in smog days. Moreover, it is not enough for retailers to demonstrate general information of products, but to optimize online exhibition by showing products in a variety of scenarios. For example, posting videos and pictures of delicious dishes made with the food in its online interface to attract consumers' attention and stimulate purchases.

Second, although our findings suggested that in-store interactive activities have a negative moderating role on the positive relationship between smog pollution and online purchase tendency, retailers should consider reducing in-store interactive activities when the smog is severe. Instead, retailers could develop those interactive activities through online channels to improve customer loyalty, reinforcing their relationship with customers. In addition, retailers can appropriately adjust layouts or designing interfaces to enhance shopping atmospheres and customer experiences. Paz and Delgado (2020) indicate that digital atmospheres are important to prompt online sales. Under the season with heavy smog pollution, retailers can change the design and layout of online platforms to help customers reduce anxiety and anxiety. For example, retailers can use designs/background colors that call to mind spring, vitality, and health, such as flowers, green grass, blue skies, and white clouds.

Third, healthy eating habits play a positive moderating role in the relationship between the degree of smog pollution and the tendency to purchase fresh food through online channels. Consumers who have higher recognition of healthy eating are more sensitive to the adverse effects of smog and care more about how to keep healthy. They tend to shop online instead of going outside on smog days. These customers are likely to buy only the products they search for specifically. During periods of heavy smog pollution, retailers can promote consumption and stimulate cross-category purchase behaviors by launching cross-category coupons, group-buying coupons and promotions for online channels (Wan et al., 2020). Foods recommended for countering the negative effects of smog can be placed in a prominent position, together with a short description of their functions and benefits. In this way, retailers can guide consumers to form a healthy anti-smog diet and promote cross-category purchasing behavior.

Our findings can also enlighten the pricing policymakers. Online channels provide an easier way to compare prices and information. Our results show that customers are more inclined to buy these price-variant products through online channels on smog day, which suggests pricing policymakers can integrate their pricing strategy with channel strategy to respond to the demand changes.

By taking an empirical approach, this paper aims to enrich the understanding of how consumer behavior is affected by the external environment. There are some inevitable shortcomings to this research owing to the difficulty of obtaining data, especially certain qualitative data. First, because certain information about consumers is withheld by merchants due to privacy concerns, the study fails to incorporate demographic characteristics such as personal income, gender, age. However, we include the individual fixed effects in the regression model, which controls individual characteristics that do not vary over time. Second, consumers' channel selection behavior may also be affected by factors such as a store's distance from home and whether consumers visit a store on their commute. Subsequent research can explore an unfavorable external environment's impact on consumers' purchasing behavior from other dimensions.

Third, since the disclosure status and the retailer's business have undergone major adjustments after 2017, our sample only includes data from 2014 to 2016. With the rapid development of e-commerce in recent years, one possible concern is whether the result derived from past data (i.e., there is a positive relationship between smog and online purchase) could remain valid. To address this concern, we retest the relationship between PM2.5 and online purchases proportion by year. These results (as shown in Table 8) indicate a consistent positive relationship between PM2.5 and the proportion of online purchases at the store-, the product- and the individual- levels for each year, which support our main findings and address the concern about possible time heterogeneity. Moreover, from 2014 to 2016, the coefficients of PM2.5 show an upward trend at the store- and individual- levels. The reason might be that a larger share of people has online shopping experience year by year, and this experience makes it easier for them to switch to online channels to buy fresh food when the external smog pollution is severer. We can reasonably infer that an unfavorable external environment would have a more significant effect in driving consumers to transfer from offline channels to online channels in recent years. Further research can consider using data from the Covid-19 period to analyze consumer shopping behavior as this period represents a more extreme and complex condition.

## Data Availability Statement

The raw data supporting the conclusions of this article will be made available by the authors, without undue reservation.

## Author Contributions

JL and JZ performed the empirical analysis. JZ provided the data. XJ collected literature and cleared data. JL wrote the first draft of the manuscript. JM did the additional tests. All authors rewrote sections of the manuscript, contributed to manuscript revision, read, and approved the submitted version.

## Conflict of Interest

The authors declare that the research was conducted in the absence of any commercial or financial relationships that could be construed as a potential conflict of interest.

## Footnotes

^1^Source: http://www.stateair.net/web/post/1/2.html.

## References

[B1] Akaah I. P. Korgaonkar P. K. Lund D. (1995). Direct marketing attitudes. J. Bus. Res. 34, 211–219. 10.1016/0148-2963(94)00119-Y

[B2] Ansari A. Mela C. F. Neslin S. A. (2008). Customer channel migration. J. Mark. Res. 45, 60–76. 10.1509/jmkr.45.1.60

[B3] Avery J. Steenburgh T. J. Deighton J. Caravella M. (2012). Adding bricks to clicks: predicting the patterns of cross-channel elasticities over time. J. Mark. 76, 96–111. 10.1509/jm.09.0081

[B4] Bakos J. Y. (1997). Reducing buyer search costs: implications for electronic marketplaces. Manag. Sci. 43, 1676–1692. 10.1287/mnsc.43.12.1676

[B5] Baumgartner H. (2002). Toward a personology of the consumer. J. Consum. Res. 29, 286–292. 10.1086/341578

[B6] Bravo L. (1998). Polyphenols: chemistry, dietary sources, metabolism, and nutritional significance. Nutr. Rev. 56, 317–333. 9838798 10.1111/j.1753-4887.1998.tb01670.x

[B7] Brunekreef B. Holgate S. T. (2002). Air pollution and health. Lancet 360, 1233–1242. 10.1016/S0140-6736(02)11274-8 12401268

[B8] Cai D. P. He Y. M. (2016). Daily lifestyles in the fog and haze weather. J. Thorac. Dis. 8, E75–E77. 10.3978/j.issn.2072-1439.2016.01.35 26904256 PMC4740148

[B9] Chaney D. Lunardo R. Bressolles G. (2016). Making the store a place of learning: the effects of in-store educational activities on retailer legitimacy and shopping intentions. J. Bus. Res. 69, 5886–5893. 10.1016/j.jbusres.2016.04.104

[B10] Chocarro R. Cortiñas M. Villanueva M. L. (2013). Situational variables in online versus offline channel choice. Electron. Commer. Res. Appl. 12, 347–361. 10.1016/j.elerap.2013.03.004

[B11] Choi S. Mattila A. S. (2009). Perceived fairness of price differences across channels: the moderating role of price frame and norm perceptions. J. Mark. Theory Pract. 17, 37–48. 10.2753/MTP1069-6679170103

[B12] Evans G. W. Jacobs S. V. (1981). Air pollution and human behavior. J. Soc. Issues 37, 95–125. 10.1111/j.1540-4560.1981.tb01059.x

[B13] Goraya M. A. S. Zhu J. Akram M. S. Shareef M. A. Malik A. Bhatti Z. A. (2020). The impact of channel integration on consumers' channel preferences: do showrooming and webrooming behaviors matter? J. Retail. Consum. Serv. 102130. 10.1016/j.jretconser.2020.102130

[B14] Hede A. M. Kellett P. (2011). Marketing communications for special events: analysing managerial practice, consumer perceptions, and preferences. Eur. J. Mark. 45, 987–1004. 10.1108/03090561111119930

[B15] Heider F. (1982). The Psychology of Interpersonal Relations. New York, NY: Psychology Press.

[B16] Hertog M. G. L. Hollman P. C. H. (1996). Potential health effects of the dietary flavonol quercetin. Eur. J. Clin. Nutr. 50, 63–71. 8641249

[B17] Heyes A. Neidell M. Saberian S. (2016). The Effect of Air Pollution on Investor Behavior: Evidence From the SandP 500 (No. w22753). Cambridge, MA: National Bureau of Economic Research.

[B18] Holmqvist J. Lunardo R. (2015). The impact of an exciting store environment on consumer pleasure and shopping intentions. Int. J. Res. Mark. 32, 117–119. 10.1016/j.ijresmar.2014.12.001

[B19] Kang H. Suh H. Yu J. (2019). Does air pollution affect consumption behavior? evidence from Korean retail sales. Asian Econ. J. 33, 235–251. 10.1111/asej.12185

[B20] Kleinlercher K. Emrich O. Herhausen D. Verhoef P. C. Rudolph T. (2018). Websites as information hubs: how informational channel integration and shopping benefit density interact in steering customers to the physical store. J. Assoc. Consum. Res. 3, 330–342. 10.1086/698415

[B21] Kleinlercher K. Linzmajer M. Verhoef P. C. Rudolph T. (2020). Antecedents of webrooming in omnichannel retailing. Front. Psychol. 11:3342. 10.3389/fpsyg.2020.606798 PMC7734289 33329282

[B22] Kollmann T. Kuckertz A. Kayser I. (2012). Cannibalization or synergy? consumers' channel selection in online–offline multichannel systems. J. Retail. Consum. Serv. 19, 186–194. 10.1016/j.jretconser.2011.11.008

[B23] Kumar P. Polonsky M. J. (2019). In-store experience quality and perceived credibility: A green retailer context. J. Retail. Consum. Serv. 49, 23–34.

[B24] Kumar V. Karande K. (2000). The effect of retail store environment on retailer performance. J. Bus. Res. 49, 167–181. 10.1016/S0148-2963(99)00005-3

[B25] Lamers S. M. Westerhof G. J. Bohlmeijer E. T. ten Klooster P. M. Keyes C. L. (2011). Evaluating the psychometric properties of the mental health continuum-short form (MHC-SF). J. Clin. Psychol. 67, 99–110. 10.1002/jclp.20741 20973032

[B26] Lawry C. A. Bhappu A. D. (2021). Measuring consumer engagement in omnichannel retailing: the mobile in-store experience (MIX) index. J. Clin. Psychol. 12:661503. 10.3389/fpsyg.2021.661503 33927671 PMC8076571

[B27] Leischnig A. Schwertfeger M. Geigenmüller A. (2011). Shopping events, shopping enjoyment, and consumers' attitudes toward retail brands—an empirical examination. J. Retail. Consum. Serv. 18, 218–223. 10.1016/j.jretconser.2010.11.002

[B28] Leszczyc P. T. P. Timmermans H. (2001). Experimental choice analysis of shopping strategies. J. Retail. 77, 493–509. 10.1016/S0022-4359(01)00054-9

[B29] Li H. Yang S. Kang H. Shi V. (2020). “Buy online, pick up in store” under fit uncertainty: to offer or not to offer. Complexity 2020:3095672. 10.1155/2020/3095672

[B30] Li J. Moul C. C. Zhang W. (2017). Hoping grey goes green: air pollution's impact on consumer automobile choices. Mark. Lett. 28, 267–279. 10.1007/s11002-016-9405-2

[B31] Li Q. Peng C. H. (2016). The stock market effect of air pollution: evidence from China. Appl. Econ. 48, 3442–3461. 10.1080/00036846.2016.113967931478171

[B32] McCoskey S. Kao C. (1998). A residual-based test of the null of cointegration in panel data. Econ. Rev. 17, 57–84. 10.1080/07474939808800403

[B33] Morton F. S. Zettelmeyer F. Silva-Risso J. (2001). Internet car retailing. J. Ind. Econ. 49, 501–519. 10.1111/1467-6451.00160

[B34] Mosquera A. Olarte-Pascual C. Ayensa E. J. Murillo Y. S. (2018). The role of technology in an omnichannel physical store: assessing the moderating effect of gender. Span. J. Mark. ESIC 22, 63–82. 10.1108/SJME-03-2018-008

[B35] Neslin S. A. Shankar V. (2009). Key issues in multichannel customer management: current knowledge and future directions. J. Interact. Mark. 23, 70–81. 10.1016/j.intmar.2008.10.005

[B36] Nicholson M. Clarke I. Blakemore M. (2002). “One brand, three ways to shop”: situational variables and multichannel consumer behaviour. Int. Rev. Retail Distrib. Consum. Res. 12, 131–148. 10.1080/09593960210127691

[B37] Oh H. Kwon K. N. (2009). An exploratory study of sales promotions for multichannel holiday shopping. Int. J. Retail Distrib. Manag. 37, 867–887. 10.1108/09590550910988048

[B38] Pauwels K. Leeflang P. S. Teerling M. L. Huizingh K. E. (2011). Does online information drive offline revenues?: only for specific products and consumer segments! J. Retail. 87, 1–17. 10.1016/j.jretai.2010.10.001

[B39] Paz M. D. R. Delgado F. J. (2020). Consumer experience and omnichannel behavior in various sales atmospheres. Front. Psychol. 11:1972. 10.3389/fpsyg.2020.01972 32849155 PMC7427578

[B40] Polyfenols B. L. (1998). Chemistry, dietary sources, metabolism, and nutritional significanve. Nutr. Rev. 56, 317–333. 10.1111/j.1753-4887.1998.tb01670.x 9838798

[B41] Power M. C. Kioumourtzoglou M. A. Hart J. E. Okereke O. I. Laden F. Weisskopf M. G. (2015). The relation between past exposure to fine particulate air pollution and prevalent anxiety: observational cohort study. BMJ 350:h1111. 10.1136/bmj.h1111 25810495 PMC4373600

[B42] Reardon J. McCorkle D. E. (2002). A consumer model for channel switching behavior. Int. J. Retail Distrib. Manag. 30, 179–185. 10.1108/09590550210423654

[B43] Sands S. Oppewal H. Beverland M. (2015). How in-store educational and entertaining events influence shopper satisfaction. J. Retail. Consum. Serv. 23, 9–20. 10.1016/j.jretconser.2014.11.004

[B44] Schröder H. Zaharia S. (2008). Linking multi-channel customer behavior with shopping motives: an empirical investigation of a German retailer. J. Retail. Consum. Serv. 15, 452–468. 10.1016/j.jretconser.2008.01.001

[B45] Smith M. F. (1999). Urban versus suburban consumers: a contrast in holiday shopping purchase intentions and outshopping behavior. J. Consum. Mark. 16, 58–73. 10.1108/07363769910250778

[B46] Turley L. W. Milliman R. E. (2000). Atmospheric effects on shopping behavior: a review of the experimental evidence. J. Bus. Res. 49, 193–211. 10.1016/S0148-2963(99)00010-7

[B47] Verhoef P. C. Neslin S. A. Vroomen B. (2007). Multichannel customer management: understanding the research-shopper phenomenon. Int. J. Res. Mark. 24, 129–148. 10.1016/j.ijresmar.2006.11.002

[B48] Wan Q. Yang S. Liao Y. Xia Y. (2020). Group-buying coupons considering consumers' perceived ease of use. Int. Trans. Oper. Res. 27, 1638–1663. 10.1111/itor.12482

[B49] Xing Y. F. Xu Y. H. Shi M. H. Lian Y. X. (2016). The impact of PM2.5 on the human respiratory system. J. Thorac. Dis. 8, E69–E74. 10.3978/j.issn.2072-1439.2016.01.19 26904255 PMC4740125

[B50] Xu X. Jackson J. E. (2019). Examining customer channel selection intention in the omni-channel retail environment. Int. J. Prod. Econ. 208, 434–445. 10.1016/j.ijpe.2018.12.009

[B51] Yang S. Lu Y. Chau P. Y. (2013). Why do consumers adopt online channel? an empirical investigation of two channel extension mechanisms. Decis. Support Syst. 54, 858–869. 10.1016/j.dss.2012.09.011

[B52] Zhang J. Mu Q. (2018). Air pollution and defensive expenditures: evidence from particulate-filtering facemasks. J. Environ. Econ. Manag. 92, 517–536. 10.1016/j.jeem.2017.07.006

